# *In Situ* Tumor Vaccine Expressing Anti-CD47 Antibody Enhances Antitumor Immunity

**DOI:** 10.3389/fonc.2022.897561

**Published:** 2022-06-28

**Authors:** Bin Zhang, Yongheng Shu, Shichuan Hu, Zhongbing Qi, Yanwei Chen, Jinhu Ma, Yunmeng Wang, Ping Cheng

**Affiliations:** State Key Laboratory of Biotherapy and Cancer Center/Collaborative Innovation Center for Biotherapy, West China Hospital, Sichuan University, Chengdu, China

**Keywords:** *in situ* tumor vaccine, oncolytic adenovirus, tumor therapy, tumor immune microenvironment, antitumor immunity, CD47

## Abstract

*In situ* tumor vaccine is a potential cancer therapy due to their advantages in induction of antitumor immune responses. Oncolytic virotherapy utilizes natural or engineered oncolytic viruses to kill tumors selectively, representing a promising *in situ* tumor vaccine for cancer immunotherapy. In addition to direct oncolysis, oncolytic viruses elicit potent and durable antitumor immune responses by induction of immunogenic cell death of tumors. Membrane protein CD47 overexpressed on tumor cells engages in “don’t eat me” signal that prevents macrophages from engulfing tumor cells. CD47-targeting agents have been tested *via* preclinical and clinical trials. As potential tumor vaccine vectors, oncolytic viruses can be engineered to express anti-CD47 antibodies to induce potentiated tumor killing. Therefore, we developed an adenovirus-based tumor vaccine loaded with a CD47-targeting nanobody fused with the IgG2a Fc protein. B16-F10 melanoma, A20 lymphoma, and 4T1 breast cancer models in immunocompetent mice were established to evaluated *in vivo* antitumor efficacy of *in situ* tumor vaccination. The tumor vaccine armed with a nanobody against CD47 induced durable suppression of the tumor and long-term survival of tumor-bearing mice, and also elevated the number of tumor-infiltrating immune cells with an activated immunophenotype, suggesting that it could remodel the tumor immune microenvironment. Systemic antitumor effects and immune memory were also observed in immunocompetent mice following *in situ* vaccination with the anti-CD47 tumor vaccines; tumorigenesis was completely inhibited in these mice after tumor re-challenge. The recombinant anti-CD47 tumor vaccine has an effectual antitumor activity and may be a promising antitumor agent.

## Introduction

*In situ* tumor vaccination induces potent antitumor immune responses by injecting immune activator such as vaccines carrying tumor antigens into tumor tissue (1). Oncolytic viruses (OVs), as popular *in situ* vaccine candidates, are a class of therapeutic viruses that naturally have or are engineered to have the capacity for tumor killing. Their antitumor effect is mainly attributed to the direct oncolysis and the induction of immune responses (2). Aberrant signaling pathways and gene expression of tumors provide OVs an advantage of selective replication and propagation within these malignant cells, which can lead to direct oncolysis without causing damage to normal tissues. After *in situ* tumor vaccination, OVs can induce immunological cell death, with tumors serving as an *in situ* tumor antigen repository, including tumor neoantigens and shared antigens (3). The direct oncolysis causes the release of soluble antigens by tumors, including tumor-associated antigens and viral antigens, and additionally, other cell populations within the tumor microenvironment (TME) also produce and release inflammatory cytokines and chemokines during oncolytic virotherapy (4, 5). These proinflammatory substances further elicit potential intrinsic and adaptive immune responses against carcinoma cells (6, 7). Currently, the United States Food and Drug Administration has approved an attenuated herpes simplex virus carrying granulocyte-macrophage colony-stimulating factor, also known as talimogene laherparepvec (Imlygic), for melanoma treatment (8). There is further scope for improving the antitumor effect of oncolytic virotherapy by identifying optimal targets and enhancing immunity regulation.

Carcinoma cells can adopt several survival strategies for evading immune surveillance by macrophages, including the overexpression of anti-phagocytic surface proteins such as CD47 (9), and CD24 (10). CD47 or integrin-associated protein is a transmembrane glycoprotein ubiquitously expressed on normal cells, and it is often aberrantly overexpressed on various solid and hematopoietic tumors (9, 11). CD47 engages in “don’t eat me” signal regulation by binding to its receptor signal regulatory protein α (SIRPα), which is expressed on all myeloid-derived immune cells, including granulocytes, monocytes, macrophages, and dendritic cells. The binding of CD47 to SIRPα leads to the phosphorylation of immunoreceptor tyrosine-based inhibitory motifs on the cytoplasmic tail of SIRPα followed by recruitment and activation of Src homology phosphatase 1 and 2, ultimately restricting phagocytosis (12).

CD47 overexpression is associated with poor prognosis in cancer patients (13). Agents targeting CD47 or its ligand SIRPα have exhibited efficacy in tumor-bearing mouse models and human trials (14, 15). OVs are ideal gene delivery vectors in cancer therapy, among which adenoviruses are popular candidates for oncolytic virotherapy owing to their low pathogenicity, high loading capacity for foreign genes, and relative ease of manufacturing. Oncolytic adenoviruses armed with therapeutic full-length monoclonal antibodies have been developed (16). The gene sequence of a full-length monoclonal antibody is generally long, and this can affect the packing and replication of the adenovirus. A nanobody is a single-domain antibody composed only of the variable region of the heavy chain and represents the minimal antigen-binding fragment with extremely high binding affinity and stability (17). Therefore, we used an oncolytic adenovirus to express a CD47-targeting nanobody to combine the advantages of enhanced phagocytosis in response to the blocking of CD47 with induction of immune responses to OVs. Moreover, given that the effects of nanobody monotherapy are mild due to absence of the Fc domain (17), we sought to insert a Fc-fusion protein to strengthen the antibody-dependent cellular phagocytosis by macrophages (18).

Herein, we constructed an adenovirus-based tumor vaccine expressing a mouse nanobody antagonist of CD47 fused with the IgG2a Fc protein (mCD47nb-Fc) and investigated its antitumor effects. The recombinant tumor vaccine armed with mCD47nb-Fc (oAd-mCD47nb-Fc) exhibited a powerful antitumor activity. oAd-mCD47nb-Fc enhanced immunological infiltration within the TME and shifted the phenotype of immune cells toward an immune-activated status. In addition to remodeling the local TME, a long-term and durable systemic antitumor immunity was observed in response to oAd-mCD47nb-Fc therapy. Importantly, the intratumoral delivery of mCD47nb-Fc by the adenovirus vector did not cause severe anemia, thrombocytopenia, and other adverse events often seen with macrophage-mediated cellular phagocytosis after the systemic administration of anti-CD47 agents.

## Materials and Methods

### Cell Culture

The murine triple-negative breast cancer cell line 4T1 (RRID: CVCL_0125), murine melanoma cell line B16-F10 (RRID: CVCL_0159), murine colon adenocarcinoma cell line MC38 (RRID: CVCL_B288), murine B cell lymphoma cell line A20 (RRID: CVCL_1940), and murine fibroblast cell line L929 (RRID: CVCL_0462) were purchased from ATCC. 4T1, B16-F10, MC38, and L929 were cultured in Dulbecco’s Modified Eagle’s medium (DMEM) supplemented with 10% fetal bovine serum (FBS). A20 cells were cultured in RPMI 1640 supplemented with 10% FBS and 5 mM β-mercaptoethanol. All cells were maintained at 37°C and 5% CO_2_. All experiments were performed with mycoplasma-free cells.

### Preparation of the Recombinant Adenovirus

An oncolytic adenovirus construct containing gene expression cassettes of E1A-IRES-E1B transgene driven by the human telomerase reserve transcriptase promoter (hTERT) was produced by using the pDC316 vector based on Gibson assembly reactions. The transgene of interest mCD47nb-Fc, under the control of the mCMV promoter, was cloned into the aforementioned oncolytic adenovirus shuttle vector using Gibson assembly reactions. HEK293A cells (RRID: CVCL_6910) were co-transfected with backbone plasmid and shuttle vector (using Ad-Max packaging system) to complete the virus packaging. Viral particles were amplified in HEK293 cells (RRID: CVCL_0045) and purified by CsCl gradient centrifugation followed by dialysis against a dialysis buffer. The purified oncolytic adenoviruses were stored at -80°C until use.

### Immunoblotting

To verify the expression of protein of interest, tumor cells were infected with oAd-mCD47nb-Fc for 48 h at a dose of MOI 20 and then were lysed by utilization of RIPA lysis buffer and 10 mM PMSF. Cell lysates were analyzed by immunoblotting.

To examine immunological cell death, tumor cells were infected with oAd-ctr or oAd-mCD47nb-Fc for 48 h at a dose of MOI 20. Media was collected, and cells were lysed by utilization of RIPA lysis buffer and 10 mM PMSF. Media and cell lysates were analyzed by immunoblotting with antibodies against HMGB1.

### Binding Assay

Tumor cell lines were plated in 24-well plates with DMEM supplemented with 2% FBS followed by infection with the oncolytic adenovirus at a dose of MOI 20. CD47 occupancy on tumor cells was tested by flow cytometry on day 2 after viral infection.

### Isolation and Culture of Bone Marrow-Derived Macrophages

Bone marrow cells were harvested from the tibia and femur of C57bl/6 or Balb/c mice. BMDMs were produced by incubating the bone marrow cells in DMEM (high glucose) supplemented with 10% FBS and 30% L929 supernatant for 6-7 days and collecting the adherent fraction by enzymatic dissociation. BMDMs were characterized by CD11b and F4/80 double positive staining.

### *In Vitro* Phagocytosis Assay

To quantify the *in vitro* phagocytosis, tumor cells were pretreated with oAd-ctr or oAd-mCD47nb-Fc for 48 h at a dose of MOI 20 followed by labeling with PKH26, and BMDMs were labeled with CFSE. BMDMs were co-cultured with tumor cells for 3 h at 37°C and 5% CO_2_. Flow cytometry and confocal fluorescence microscopy were utilized to examine phagocytosis of tumors by BMDMs. Phagocytosis was quantified as the percentage of CFSE-positive macrophages that have engulfed PHK26-positive tumor cells.

### Animal Models

Mice were randomly assigned into various groups. For the B16-F10 or A20 mouse model, 1×10^6^ B16-F10 cells or 4T1 cells, or 5×10^6^ A20 cells were subcutaneously inoculated on a single flank of C57bl/6 mice or Balb/c mice, respectively. All mice were female mice aged 6–8 weeks. adenovirus-based tumor vaccines or PBS were intratumorally injected at a dose of 5×10^8^ pfu per mouse when the tumors reached approximately 75-120 mm^3^ and then once every 6 days for a total of three times. For the A20 rechallenge model, 5×10^6^ A20 cells were again subcutaneously implanted on the flank of mice whose A20 tumors were eliminated completely on day 90 after first tumor implantation. Naïve mice were also similarly subjected to the challenge. All animal experiments were approved by the Institutional Animal Care and Use Committee of Sichuan University, Chengdu, China.

### Flow Cytometry

Mice were sacrificed on the indicated day after treatment. For preparation of single-cell solutions from tumor or spleen tissue, tumor tissue was digested with collagenase IV for 30 min at 37°C followed by washing with PBS. Splenocytes were released from the spleen tissue by passing through a 70-µm cell strainer, washing with PBS, and removing red blood cells by using an RBC lysis buffer. Dead cells were excluded by staining with Fixable Viability dye. Extracellular antibodies including anti-CD3, anti-CD4, anti-CD8, anti-PD1, anti-CD69, anti-CD45, anti-CD11b, anti-F4/80, anti-Gr1, anti-CD206, anti-MHC II, anti-CD44, and anti-CD62L antibodies were used to measure the expression levels of membrane surface proteins. Cells were fixed and permeabilized by using Intracellular Staining Fixation and Permeabilization Buffer following extracellular staining. Intracellular antibodies including anti-INOS, anti-ARG1, anti-IFNγ, and anti-TNFα antibodies were used to assess the production of intracellular proteins. For the assessment of production of IFNγ and TNFα by T cells of spleens, T cells were harvested from spleens by using murine lymphocyte separation medium and stimulated for 2 h with 50 ng/ml PMA and 100 µg/ml ionomycin in the presence of brefeldin A.

### Hematoxylin and Eosin (H&E) Staining

4T1 breast tumor-bearing mice and B16-F10 melanoma bearing-mice were sacrificed on the indicated day after initial treatment. Tumor tissues were fixed by using 4% paraformaldehyde, and then the paraffin-embedded were stained with H&E according to standard methods.

### Immunohistochemistry

4T1 breast tumor-bearing mice were sacrificed on the indicated day after initial treatment. Tumor tissues were fixed by using 4% paraformaldehyde, and then the paraffin-embedded sections were incubated with primary CD3 antibody, secondary biotin-conjugated goat anti-rabbit antibody, streptavidin-conjugated horseradish peroxidase, and DAB.

### Routine Blood Examination

B16-F10 melanoma-bearing mice were intratumorally injected with PBS or adenovirus-based tumor vaccines at a dose of 5×10^8^ pfu per mouse. On day 2 after the first treatment, the mice were anesthetized, and blood samples were withdrawn from the retro-orbital sinus and subjected to routine blood test.

### Statistical Analysis

All values were statistically analyzed using GraphPad Prism v.8.0 and are presented as the mean ± SEM. The results were compared with one-way ANOVA or two-tailed unpaired *t* test. The survival curves of mice were analyzed by the log-rank (Mantel-Cox) test. All individual values were obtained from different samples.

## Results

### oAd-mCD47nb-Fc Enhances the Phagocytic Function of Macrophages and Induces Immunological Cell Death *In Vitro*

**Figure 1:**
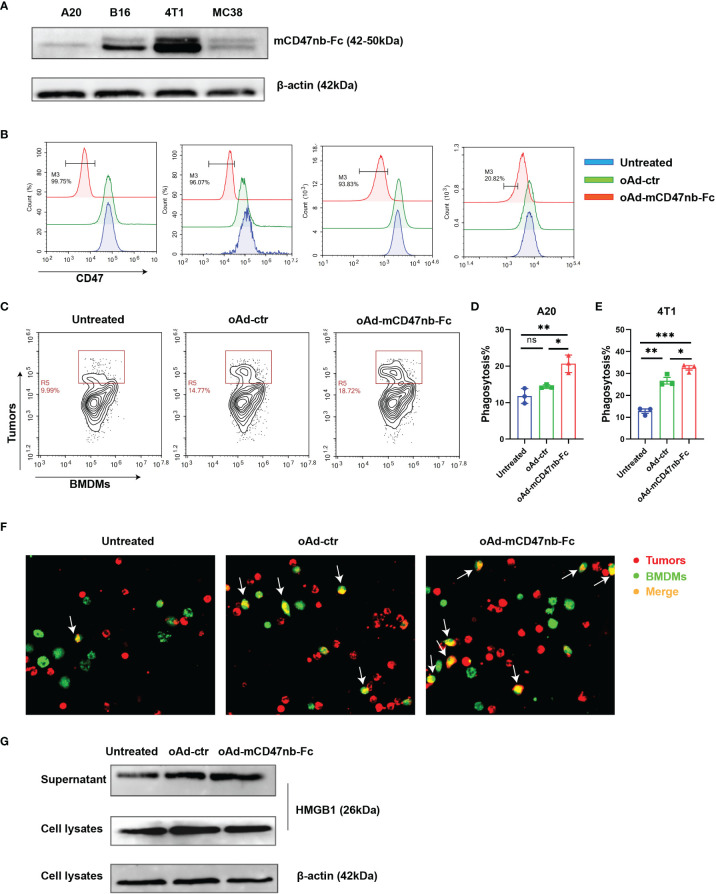
oAd-mCD47nb-Fc enhances the phagocytic function of macrophages and induces immunological cell death *in vitro*. **(A)** Expression of mCD47nb-Fc in different tumor cells at 48 h after infection with oAd-mCD47nb-Fc at a dose of MOI 20. **(B)** The CD47-blocking function of oAd-mCD47nb-Fc on various tumor cell lines examined. **(C, D)** Representative flow cytometry images **(C)** and the quantification **(D)** of phagocytosis of A20 cells by BMDMs in response to different treatments. **(E)** Phagocytosis of 4T1 cells by BMDMs in response to different treatments. Phagocytosis was quantified as the percentage of CFSE-positive macrophages that have engulfed PHK26-positive A20 or 4T1 cells. **(F)** Representative fluorescent images showing phagocytosis of A20 cells by BMDMs. **(G)** Western blot analysis detecting HMGB1 expression in A20 conditioned media and cell lysates at 48 h post virus infection at a dose of MOI 20. Data are representative of three independent experimental replicates and shown as mean ± SEM. *P ≤ 0.05, **P ≤ 0.01, ***P ≤ 0.001, ns, not significant (P>0.05), all values were compared using one-way ANOVA with Tukey’s multiple comparison test.

To enhance tumor tropism, the *E1A* and *E1B* genes of the adenovirus were controlled by hTERT. We constructed a tumor-selective type 5 oncolytic adenovirus armed with mCD47nb-Fc and a control adenovirus without carrying genes of interest (oAd-ctr) (**Supplemental Information Figure S1A**). The results from western blot showed that tumor cells expressed protein of interest mCD47nb-Fc after oAd-mCD47nb-Fc infection, although the amount of protein expression was different among different cells (**Figure 1A**). Multiple tumor cells have developed the ability to evade phagocytosis by phagocytes through overexpression of the surface protein CD47, and consistent with this, high CD47 expression was observed on several carcinoma cell lines examined (**Supplemental Information Figure S1B**). To verify whether the mCD47nb-Fc proteins released by the oncolytic adenovirus vector have a high binding affinity to CD47 on tumors, we tested CD47 occupancy after viral infection by flow cytometry and found that oAd-mCD47nb-Fc could effectively block CD47 in contrast to its counterpart (**Figure 1B**). Next, the ability of macrophages for phagocytic function was tested in response to oAd-mCD47nb-Fc treatment. BMDMs from Balb/c mice were isolated and stained with CFSE followed by co-culture with tumor cells that were pretreated with the oncolytic adenovirus or PBS and labeled with the red fluorescent dye PKH26. Flow cytometry was used to examine the phagocytosis of tumors by BMDMs, as a result, BMDMs showed highest phagocytic ability to oAd-mCD47nb-Fc-infected A20 cells compared to baseline and that in the oAd-ctr group (**Figures 1C, D**). Similarly, the phagocytic ability of macrophages to oAd-mCD47nb-Fc-treated 4T1 cells was also significantly highest (**Figure 1E**, **Supplemental Information Figure S1C**). The result from confocal fluorescence microscopy was consistent with aforementioned results (**Figure 1F**), indicating that CD47 inhibition is necessary for enhancing phagocytosis by macrophages. Additionally, a dose-dependent toxicity was observed in tumor cells after infection with oncolytic viruses (**Supplemental Information Figure S1D**). Tumor cells were also induced to release a large amount of HMGB1 in conditioned media after oAd-mCD47nb-Fc or oAd-ctr infection (**Figure 1G**). Overall, oAd-mCD47nb-Fc infection can block CD47 and enhance phagocytosis through CD47 inhibition, as well as inducing immunological cell death.

### *In Situ* Tumor Vaccination With oAd-mCD47nb-Fc Represses Tumor Growth in Murine Models

**Figure 2:**
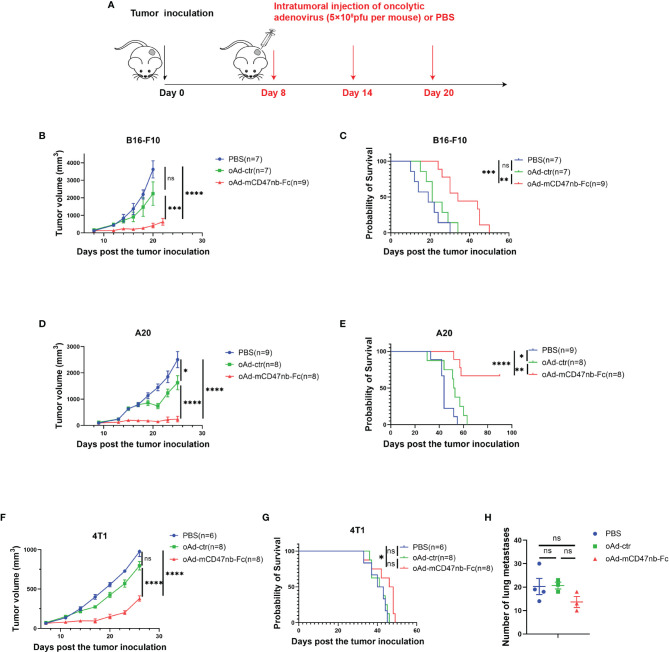
*In situ* tumor vaccination with oAd-mCD47nb-Fc represses tumor growth in murine models. **(A)** Treatment schedule. C57bl/6 or Balb/c mice were inoculated subcutaneously with 1×10^6^ B16-F10 cells, or 4T1 cells, or 5×10^6^ A20 cells, respectively on a hind flank. When the tumor volume reached approximately 120 mm^3^, 75 mm^3^, or 100 mm^3^, respectively, the mice were intratumorally administered PBS or adenovirus-based tumor vaccines at a dose of 5×10^8^ pfu per mouse every 6 days for a total of three times. **(B)** Average tumor growth curves of B16-F10 tumor-bearing mice in various groups. **(C)** Kaplan–Meier survival curves for B16-F10 tumor-bearing mice. **(D)** Average tumor growth curves of A20 tumor-bearing mice under the treatment of PBS, oAd-ctr, or oAd-mCD47nb-Fc. **(E)** Kaplan–Meier survival curves for A20 tumor-bearing mice. **(F)** Average tumor growth curves of 4T1 tumor-bearing mice in various groups. **(G)** Kaplan–Meier survival curves for 4T1 tumor-bearing mice. **(H)** Quantification of the number of lung metastatic foci in mice treated with PBS, oAd-ctr, or oAd-mCD47nb-Fc. *P ≤ 0.05, **P ≤ 0.01, ***P ≤ 0.001, ****P<0.0001, ns, not significant (P>0.05). Comparisons were made using one-way ANOVA with Tukey’s multiple comparison test (b, d, f and h) and Log-rank (Mantel–Cox) test **(C, E, G)**. All data are shown as mean ± SEM.

To explore the *in vivo* therapeutic effects, we established B16-F10 tumor-bearing mouse model and administered PBS or adenovirus-based tumor vaccines intratumorally when the tumor size reached approximately 120 mm^3^ (**Figure 2A**). Injection of oAd-ctr induced a slight antitumor effect in B16-F10 melanoma murine model; however, administration of oAd-mCD47nb-Fc significantly suppressed melanoma progression and prolonged the survival of mice (**Figures 2B, C**). A subcutaneous lymphoma model was also established by using the A20 cell line. Initiating therapy with PBS, oAd-ctr or oAd-mCD47nb-Fc when tumor size reached approximately 100 mm^3^. Treatment with oAd-ctr resulted in a relatively significant tumor growth inhibition and increased the survival of mice compared to PBS control, but it did not result in complete tumor eradication. Intriguingly, 62.5% mice (five out of eight) achieved complete response with oAd-mCD47nb-Fc therapy, and these mice survived over 90 days since tumor inoculation (**Figures 2D-E**). We also assessed the efficacy of oAd-mCD47nb-Fc in 4T1 mouse breast cancer model. Initiating therapy with PBS, oAd-ctr or oAd-mCD47nb-Fc when tumor size reached approximately 75 mm^3^. Tumor growth at the injection site was inhibited with oAd-mCD47nb-Fc treatment compared to that with oAd-ctr or PBS treatment (**Figure 2F**). However, the extent of tumor suppression with oAd-mCD47nb-Fc did not significantly prolong survival (**Figure 2G**), which may be linked to the significant lung metastasis of 4T1 carcinoma. Indeed, the number of lung metastatic foci was not significantly different among the three groups (**Figure 2H**), which suggested that oAd-mCD47nb-Fc monotherapy has limited effects against lung metastasis of 4T1 carcinoma. Collectively, although oAd-mCD47nb-Fc monotherapy did not inhibit tumor metastasis, it still had some therapeutic effect on injected tumors.

### oAd-mCD47nb-Fc Enhances Immune Infiltration Within the TME

**Figure 3:**
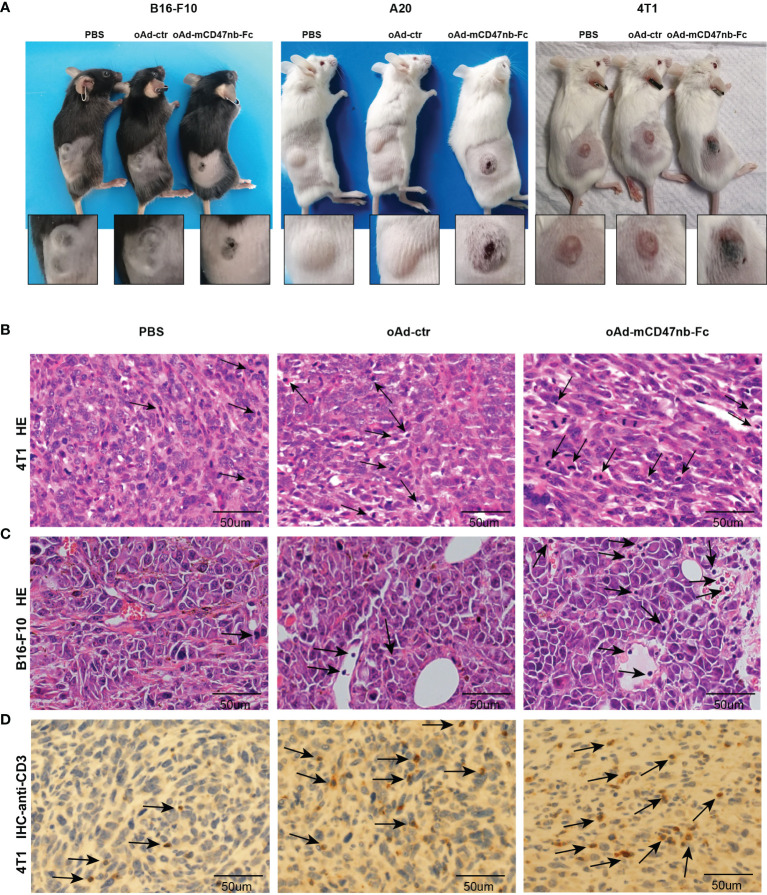
oAd-mCD47nb-Fc enhances immune infiltration within the TME. **(A)** Representative photographs of tumor scabbing and tissue swelling in tumor-bearing mice after treatment with oAd-mCD47nb-Fc. **(B)** Representative HE staining image of 4T1 breast tumor tissue of mice treated with PBS, oAd-ctr, or oAd-mCD47nb-Fc. **(C)** Representative HE staining image of B16-F10 melanoma tissue of mice treated with PBS, oAd-ctr, or oAd-mCD47nb-Fc. **(D)** Representative anti-CD3 immunohistochemical staining image of 4T1 breast tumor tissue of mice treated with the indicated agent. Black arrows indicate immune cells.

oAd-mCD47nb-Fc potently reduced the growth of established tumors in aforementioned models, and intriguingly, at the same time, we observed marked scabbing at the core of the tumor mass together with tissue swelling at the periphery of the tumor after 2–3 days of treatment with oAd-mCD47nb-Fc (**Figure 3A**). The swelling was temporary and lasted for about a week, whereas the tumor scab lasted for a longer time. In contrast, such manifestations were not noted in either oAd-ctr or PBS treatment group. The salient scabbing and swelling were indicative of a strong inflammatory response at the tumor site; thus, we sought to identify the immune landscape of the TME and explore how host immunity engages in antitumor activity. On day 10 following the first administration, 4T1 tumors were harvested, and the sections were fixed and subjected to HE staining. Enhanced immunological infiltration was observed in the TME of oAd-mCD47nb-Fc-treated mice (**Figure 3B**). Additionally, we observed similar results in B16-F10 melanoma models. In the group of oAd-mCD47nb-Fc treatment, increased immune cells were seen in and surrounding the blood vessels (**Figure 3C**). We performed immunohistochemical staining against CD3, as a result, increased CD3^+^ T lymphocytes were observed in response to oAd-mCD47nb-Fc or oAd-ctr treatment (**Figure 3D**).

### oAd-mCD47nb-Fc Reprograms Tumor-Associated Macrophages

**Figure 4:**
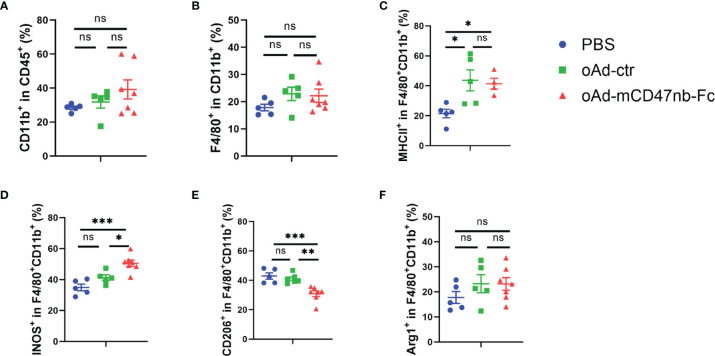
oAd-mCD47nb-Fc reprograms tumor-associated macrophages (TAMs). C57bl/6 mice were implanted subcutaneously with B16-F10 melanoma cells on a hind flank, and were administered PBS or adenovirus-based tumor vaccines intratumorally when the tumor volume reached approximately 120 mm^3^. Mice were sacrificed, and the tumors of mice were harvested on the day 10 after the first administration. Flow cytometry analysis of tumor-infiltrating immune cells in B16-F10 melanoma tissue. Frequency of CD11b^+^myeloid immune cells **(A)** and TAMs **(B)** within the TME after treatment with PBS, oAd-ctr, or oAd-mCD47nb-Fc. Quantification of the expression of MHCII **(C),** iNOS **(D)**, CD206 **(E)**, Arg1 **(F)** on TAMs. *P ≤ 0.05, **P ≤ 0.01, ***P ≤ 0.001, ns, not significant (P>0.05), all values were compared using one-way ANOVA with Tukey’s multiple comparison test. All data are shown as mean ± SEM.

The CD47/SIRPα axis plays a pivotal role in the regulation of macrophage functions; hence, we next assessed how oAd-mCD47nb-Fc affects TAMs. We observed that treatment with either oAd-ctr or oAd-mCD47nb-Fc led to a slight increase in the frequency of CD11b-positive myeloid immune cells in contrast to PBS control, although there was no significant difference (**Figure 4A**). Moreover, the proportion of TAMs gating on F4/80^+^ CD11b^+^ cells to CD11b^+^ cells did not significantly differ among the three groups (**Figure 4B**). Remodeling of immunosuppressed macrophages promotes antitumor activity (19). Indeed, in this study, although treatment with oAd-mCD47nb-Fc did not markedly decrease the numbers of TAMs, it shifted the phenotype of TAMs toward an immune-activated status (**Figures 4C-F**). MHC class II molecules and iNOS are generally used to characterize proinflammatory and anti-tumorigenic macrophages. Our study demonstrated that oAd-ctr treatment significantly increased the expression of MHC II by TAMs, whereas oAd-mCD47nb-Fc significantly increased the level of both MHC II and iNOS on macrophages in comparison to PBS (**Figures 4C, D**). CD206 is generally considered to be an indicator of pro-tumorigenic M2-type macrophages, and its expression on TAMs was decreased by the administration of oAd-mCD47nb-Fc (**Figure 4E**). However, the levels of Arg1 produced by TAMs did not significantly differ among the three groups (**Figure 4F**). Collectively, oAd-mCD47nb-Fc treatment could remodel TAMs by decreasing immunosuppression-polarized TAMs and increasing activation-polarized TAMs.

### oAd-mCD47nb-Fc Stimulates T Lymphocytes Within the TME and Spleen

**Figure 5:**
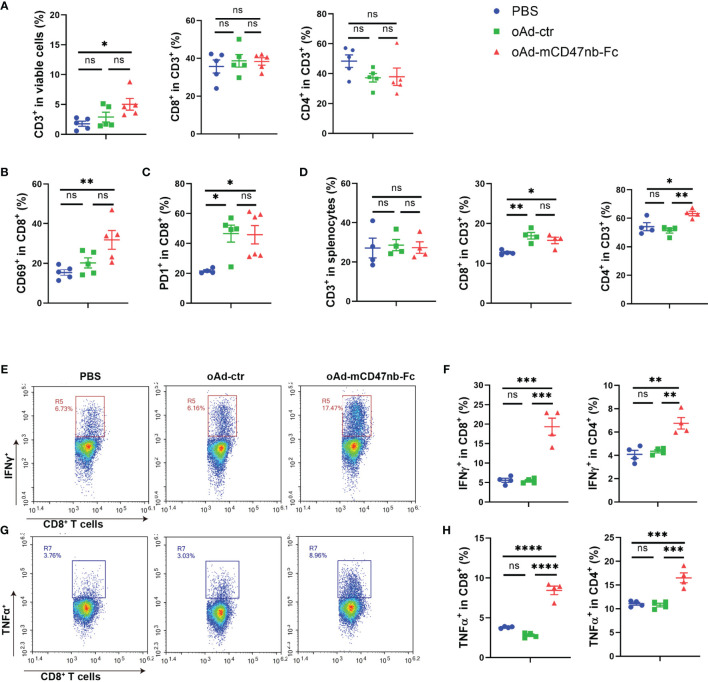
oAd-mCD47nb-Fc stimulates T lymphocytes within the TME and spleen. Balb/c mice were subcutaneously implanted with 5×10^6^ A20 lymphoma cells on the hind flank. Mice were intratumorally administered PBS or adenovirus-based tumor vaccines when the tumor volume reached approximately 100 mm^3^. Mice were sacrificed, and the tumors or spleens of mice were harvested on day 10 after the first administration. **(A)** Frequency of CD3, CD8 and CD4 positive T lymphocytes within the TME. **(B)** The expression levels of CD69 in tumor-infiltrating CD8^+^ T cells. **(C)** The expression levels of PD1 in tumor-infiltrating CD8^+^ T cells. **(D)** Frequency of CD3, CD8 and CD4 positive T lymphocytes in the spleen. **(E)** Representative plots of IFNγ levels produced by CD8^+^ T cells in the spleens of A20-bearing mice. **(F)** Expression of IFNγ by CD8^+^ T cells and CD4^+^ T cells was determined. **(G)** Representative plots of TNFα levels produced by CD8^+^ T cells in the spleens of A20-bearing mice. **(H)** Expression of TNFα by CD8^+^ T cells and CD4^+^ T cells was determined. *P ≤ 0.05, **P ≤ 0.01, ***P ≤ 0.001, ****P<0.0001, ns, not significant (P>0.05), all values were compared using one-way ANOVA with Tukey’s multiple comparison test. All data are shown as mean ± SEM.

T lymphocyte-induced immune responses are indispensable for tumor repression. Cytotoxic T lymphocytes exhibit powerful and durable tumor killing. In the B16-F10 mouse model, the frequency of CD8^+^ T cells was no significant difference among the mice injected with oAd-ctr, oAd-mCD47nb-Fc, or PBS (**Supplemental Information Figure S2A**). Additionally, both expression levels of CD69 and PD1 in tumor-infiltrating CD8^+^ T cells showed no marked difference among PBS, oAd-ctr or oAd-mCD47nb-Fc-treated mice (**Supplemental Information Figures S2B, C**). Next, a subcutaneous A20 lymphoma mouse model was established to assess whether oAd-mCD47nb-Fc has broader applicability in stimulating of antitumor immunity. In this model, the administration of oAd-mCD47nb-Fc significantly promoted CD3^+^ lymphocyte infiltration in the tumor, in which the ratio of CD3^+^ population to viable cells was approximately 2 folds and 3 folds higher than that seen with treatment with oAd-ctr and PBS, respectively (**Figure 5A**). Yet, the proportion of CD8^+^ or CD4^+^ T cells in the CD3^+^ population did not significantly differ among the three groups (**Figure 5A**). oAd-mCD47nb-Fc significantly upregulated CD69 expression on tumor-infiltrating CD8^+^ T cells in comparison to counterpart (**Figure 5B**). Additionally, treatment with either oAd-ctr or oAd-mCD47nb-Fc increased the expression of immune checkpoint molecule PD1 on tumor-infiltrating CD8^+^ T cells (**Figure 5C**). Next, we sought to examine whether oAd-mCD47nb-Fc could induce potent systemic adaptive immune responses. Spleens of A20 lymphoma-bearing mice were harvested on the indicated day after administration. Flow cytometry data suggested that the frequency of CD8^+^ CD3^+^ and CD4^+^ CD3^+^T lymphocytes was significantly higher in mice treated with oAd-mCD47nb-Fc than in mice treated with PBS or oAd-ctr (**Figure 5D**). Intriguingly, both CD4^+^ and CD8^+^ T lymphocytes could produce abundant amount of IFNγ and TNFα post oAd-mCD47nb-Fc therapy compared to PBS or oAd-ctr (**Figures 5E-H**), suggesting that oAd-mCD47nb-Fc could better activate the systemic immune. Collectively, in our study, either oAd-ctr or oAd-mCD47nb-Fc could attract and activate cytotoxic T lymphocytes to varying degrees; however, oAd-mCD47nb-Fc had better immune-stimulation potency. oAd-mCD47nb-Fc enhanced the accumulation of cytotoxic T cells with an active immunophenotype in tumors. Additionally, it induced the abundant production of IFNγ and TNFα by CD4^+^ and CD8^+^ T cells in the spleen.

### oAd-mCD47nb-Fc Protects Mice From Tumor Re-Challenge

**Figure 6:**
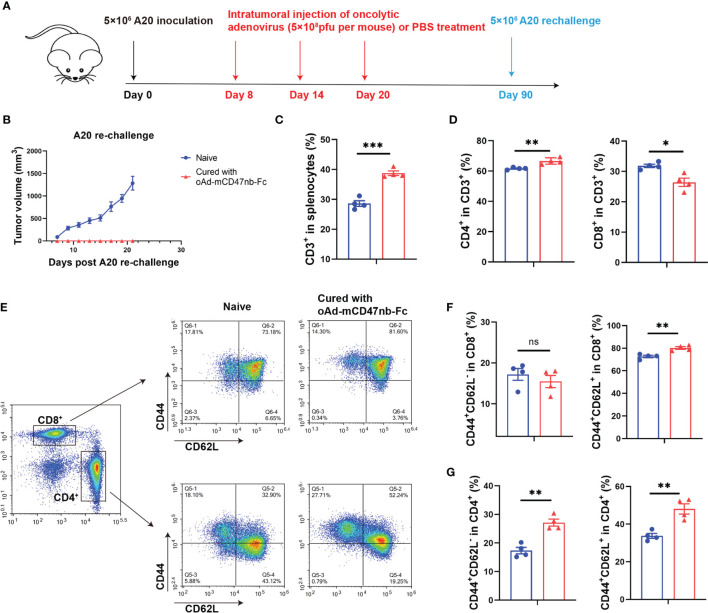
oAd-mCD47nb-Fc protects mice from tumor re-challenge. **(A)** Treatment schedule. Female Balb/c mice were subcutaneously inoculated with 5×10^6^ A20 cells on a hind flank. When the tumor volume reached 100 mm^3^, the mice were intratumorally administered PBS or adenovirus-based tumor vaccines at a dose of 5×10^8^ pfu per mouse every 6 days for a total of three times. Mice whose A20 tumor was eliminated completely were re-challenged with 5×10^6^ A20 on day 90 after implantation. Naïve mice were subjected to the challenge likewise. **(B)** Tumor growth curves of A20 rechallenged mice. **(C–G)** Analyses of immune cells of the spleens of cured and naïve mice on day 25 following re-challenge. Frequency of CD3^+^ **(C)** and CD4^+^ CD3^+^ and CD8^+^ CD3^+^ T lymphocytes **(D)** in the spleens. **(E)** Representative plots of Tem and Tcm of CD8^+^ and CD4^+^ T cells in spleens. Analyses of the frequency of Tem and Tcm of CD8^+^ **(F)** and CD4^+^ T cells **(G)** in spleens. *P ≤ 0.05, **P ≤ 0.01, ***P ≤ 0.001, ns, not significant (P>0.05). All values were compared using two-tailed unpaired t-test. All data are shown as mean ± SEM.

To completely eradiate the tumor and avoid relapse, a durable antitumor effect and immune memory are necessary. As aforementioned, oAd-mCD47nb-Fc treatment in A20 lymphoma mice resulted in 62.5% mice (five out of eight) achieving complete response (**Figure 2E**). Next, these mice exhibiting complete tumor eradiation were re-challenged against A20 lymphoma on day 90 after inoculation (**Figure 6A**). All naïve mice developed an obviously visible tumor nodule subcutaneously on the inoculation side a week after implantation; however, none of the cured mice developed a tumor until the end of the study (**Figure 6B**), suggesting that immune memory protected these mice from the tumor re-challenge. Thus, we next sought to identify the profile of memory immune cells. Mice were sacrificed on day 25 after tumor re-challenge, and the spleens of mice were harvested and examined for changes in memory immune cells by flow cytometry. The number of CD3^+^ immune cells was significantly higher in the cured group than in the naïve group (**Figure 6C**), and this increase was mainly linked to the elevated CD4^+^ lymphocytes in the cured mice (**Figure 6D**). Further investigation of the memory immunophenotype of CD8^+^ or CD4^+^ lymphocytes (**Figure 6E**) revealed a markedly enhanced frequency of either CD44^+^CD62L^-^ lymphocytes (also known as effector memory T cells, Tem) or CD44^+^CD62L^+^ lymphocytes (also known as central memory T cells, Tcm) in the population of CD4^+^ lymphocytes in cured mice (**Figure 6G**). However, in the population of CD8^+^ lymphocytes, Tem levels between the cured and naïve group did not differ significantly (**Figure 6F**).

### Intratumoral Delivery of oAd-mCD47nb-Fc Avoids Severe Systemic Toxicity

Anemia is the most common adverse event after treatment with CD47-blocking drugs due to the broad expression of CD47 on red blood cells and platelets (20). Ingram and colleagues reported that systemic toxicity including severe anemia and even death in treated mice is induced in response to the anti-CD47 nanobody infused with a IgG2a Fc (21). Therefore, we sought to assess whether oAd-mCD47nb-Fc can lead to severe anemia and other adverse events. We established B16-F10 melanoma murine models and then conducted routine blood tests on day 2 after administration of adenovirus-based tumor vaccines at a dose of 5×10^8^ pfu per mouse. The number of white blood cells and platelets did not differ significantly among mice treated with PBS, oAd-ctr, or oAd-mCD47nb-Fc (**Supplemental Information Figures S3A, B**). Although the hematocrit in mice treated with oAd-mCD47nb-Fc was decreased, the number of red blood cells and the content of hemoglobin did not differ significantly among the three groups (**Supplemental Information Figures S3C, D**). Additionally, the bodyweight of treated mice did not fluctuate significantly in the week following intratumoral injection (**Supplemental Information Figure S3E**). Collectively, these data suggested that intratumoral administration of oAd-mCD47nb-Fc at an appropriate dose does not lead to severe anemia in mice.

## Discussion

The CD47/SIRPα axis has attracted the interests of several oncologists. Potent antitumor activity was observed in response to humanized anti-SIRPα antibody hAB21combined with rituximab or PD-1/PD-L1 blockade in tumor-bearing mice (22). The therapeutic value of CD47-targeting agents has also been verified in preclinical and clinical trials. For instance, a first-in-human phase I trial of Hu5F9-G4, a humanized anti-CD47 monoclonal antibody, showed promising activity in advanced solid tumors and lymphomas (23). However, anemia, thrombocytopenia, and other adverse events caused by macrophage-mediated cellular phagocytosis limit the feasibility of the systemic administration of anti-CD47 agents (20). Here, in this study, we used a replication-selective oncolytic adenovirus to intratumorally deliver a CD47-targeting nanobody for reducing off-tumor toxicity and enhancing therapeutic efficacy. As expected, the local injection of oAd-mCD47nb-Fc did not elicit acute adverse events associated with reduced activity and weight loss, and at the same time, it also did not induce severe anemia. However, considering that the difference between murine cells and human cells, it is essential to evaluate the safety of an oncolytic adenovirus expressing human CD47 nanobody infused with the IgG2a Fc protein in humanized murine model bearing human tumors.

In B16-F10 melanoma and A20 lymphoma murine models, *in situ* vaccination with oAd-mCD47nb-Fc exhibited good antitumor activity at the injection sites, but the monotherapy could not inhibit tumor metastasis and invasion as demonstrated in the 4T1 breast cancer model. This difference in efficacy may be linked to the difference in the sensitivity of tumor models to oAd-mCD47nb-Fc therapy. This also suggests that oAd-mCD47nb-Fc monotherapy may have limited efficacy; hence, combination therapy can be considered to enhance antitumor efficacy. There have been reports confirming the antitumor activity of oncolytic viruses in combination with other antitumor modalities (24–26). In our study, oncolytic adenoviruses were demonstrated to increase expression level of PD-1 on tumor-infiltrating T lymphocytes. Upregulation of immune checkpoint expression is beneficial to the treatment of immune checkpoint inhibitors. Therefore, it may be beneficial to enhance the antitumor efficacy of oncolytic adenoviruses when administered in combination with PD-1 inhibitors, which is a focus of our follow-up research. oAd-mCD47nb-Fc induce systemic antitumor activity to some extent. Analysis of lymphocytes in the spleens of A20-bearing mice suggested that local injection of oAd-mCD47nb-Fc could elicit systemic immune responses manifested as an elevated proportion of immune-activated T lymphocytes in the spleen.

Anti-CD47 treatment was reported to decrease the proportion of intratumoral macrophages and promote the remodeling of macrophages (27). TAMs are linked to tumor progression and poor prognosis, but the depletion of TAMs comes at the expense of loss of their phagocytic and antigen-presenting functions; thus, the reprogramming of TAMs could serve as a more powerful strategy to enhance antitumor efficacy. For instance, targeting TAM activation using an agonist CD40 antibody was effective against pancreatic carcinoma (28). In our study, although oAd-mCD47nb-Fc did not significantly alter the number of macrophages within the TME, it did increase the proportion of proinflammatory macrophages and reduce anti-inflammatory macrophages.

*In situ* tumor vaccination with oAd-mCD47nb-Fc induced significant immunological infiltration of tumors, in particular eliciting a CD8^+^ T cell immune response. Similarly, oAd-ctr treatment enhanced the recruitment of immune cells, but oAd-mCD47nb-Fc significantly increased the frequency of tumor-infiltrating immune cells with an active immunophenotype. The immunogenic cell death of tumors induced by oncolytic virotherapy results in the generation of a tumor-specific immune response (29). The selective replication of OVs in tumors induces the secretion of proinflammatory cytokines and chemokines, and at the same time, the direct lysis of tumors by OVs leads to the release of soluble tumor antigens, which promote the recruitment and activation of innate and adaptive immune cells. Previous studies reported that disruption of the CD47/SIRPα axis not only enhances the phagocytosis of tumors by macrophages but also enhances DC cross-priming and triggers T cell immune responses (30–32). Here, we found that oAd-mCD47nb-Fc could not only reprogram TAMs to activate innate immunity, but also elicit a CD8^+^ T cell immune response. Many studies have confirmed that oncolytic viruses could activate CD8^+^ T cells and produce adoptive antitumor immune responses (33, 34). However, in our study, whether DC is involved in cross-priming and activating CD8^+^ T cell immune response after CD47 blockade needs further study. In addition to the induction of antitumor immunity, Martinez-Torresa and colleagues showed that programmed cell death could be induced in response to an agonist peptide of CD47 *via* phospholipase C-γ1 activation (35); hence, we speculate that interruption of the CD47/SIRPα axis may also activate other pathways of cell death in addition to enhancing phagocytosis, but in-depth investigation is necessary.

*In situ* vaccination with oAd-mCD47nb-Fc also elicited a long-term antitumor immunity, which protected mice from tumor re-challenge. The proportion of Tem in the CD4^+^ T lymphocyte population was significantly enhanced in response to oAd-mCD47nb-Fc. This verifies the supporting role of CD4^+^ T lymphocytes in the function of CD8^+^ T lymphocytes. Zuazo et al. confirmed the contribution of systemic CD4 immunity in cancer immunotherapy; according to them, CD8^+^ T cells only function effectively in patients with good CD4 immunity (36). Therefore, following a second antigen recognition, memory CD4^+^ T cells undergo rapid cloning and proliferation to promote tumor-specific CD8 immunity, which protects the host from tumors.

In this study, we used the intratumoral route of drug injection as for most oncolytic virotherapies. The local delivery of oAd-mCD47nb-Fc bypasses the neutralizing antibodies present in the circulatory system and increases the intratumoral concentration of the agents, avoiding severe anemia caused by the systemic administration of anti-CD47 monoclonal antibodies. Collectively, the adenovirus-based tumor vaccines carrying mCD47nb-Fc has a potent antitumor effect by inducing powerful and durable immune responses.

## Data Availability Statement

The original contributions presented in the study are included in the article/**Supplementary Material**. Further inquiries can be directed to the corresponding author.

## Ethics Statement

The animal study was reviewed and approved by Institutional Animal Care and Use Committee of Sichuan University.

## Author Contributions

BZ carried out experimental scheme design, experiment execution, data analysis and manuscript writing. PC provided constructive guidance, critical advice, and adequate funding, and was responsible for writing-review and editing. YS, SH, YC, ZQ, JM, and YW offered necessary help to the research. All authors contributed to the article and approved the submitted version.

## Funding

This work was supported by the National Science and Technology Major Projects of New Drugs (2018ZX09201018-013), the National Natural Science Foundation of China (81101728), Sichuan Regional Innovation Cooperation Project (20QYCX0100), the Innovation Spark Project of Sichuan University (2018SCUH0084).

## Conflict of Interest

The authors declare that the research was conducted in the absence of any commercial or financial relationships that could be construed as a potential conflict of interest.

## Publisher’s Note

All claims expressed in this article are solely those of the authors and do not necessarily represent those of their affiliated organizations, or those of the publisher, the editors and the reviewers. Any product that may be evaluated in this article, or claim that may be made by its manufacturer, is not guaranteed or endorsed by the publisher.
