# Supplementary material for: In Situ Tumor Vaccine Expressing Anti-CD47 Antibody Enhances Antitumor Immunity
Source: Front Oncol. 2022 Jun 28;12:897561. doi: 10.3389/fonc.2022.897561 (PMC9273963; doi:10.3389/fonc.2022.897561)
Supplement: Supplementary file 1 [file DataSheet_1.pdf]

In situ tumor vaccine expressing anti-CD47 antibody enhances antitumor immunity

Bin Zhang, Yongheng Shu, Shichuan Hu, Zhongbing Qi, Yanwei Chen, Jinhu Ma, Yunmeng Wang, Ping Cheng

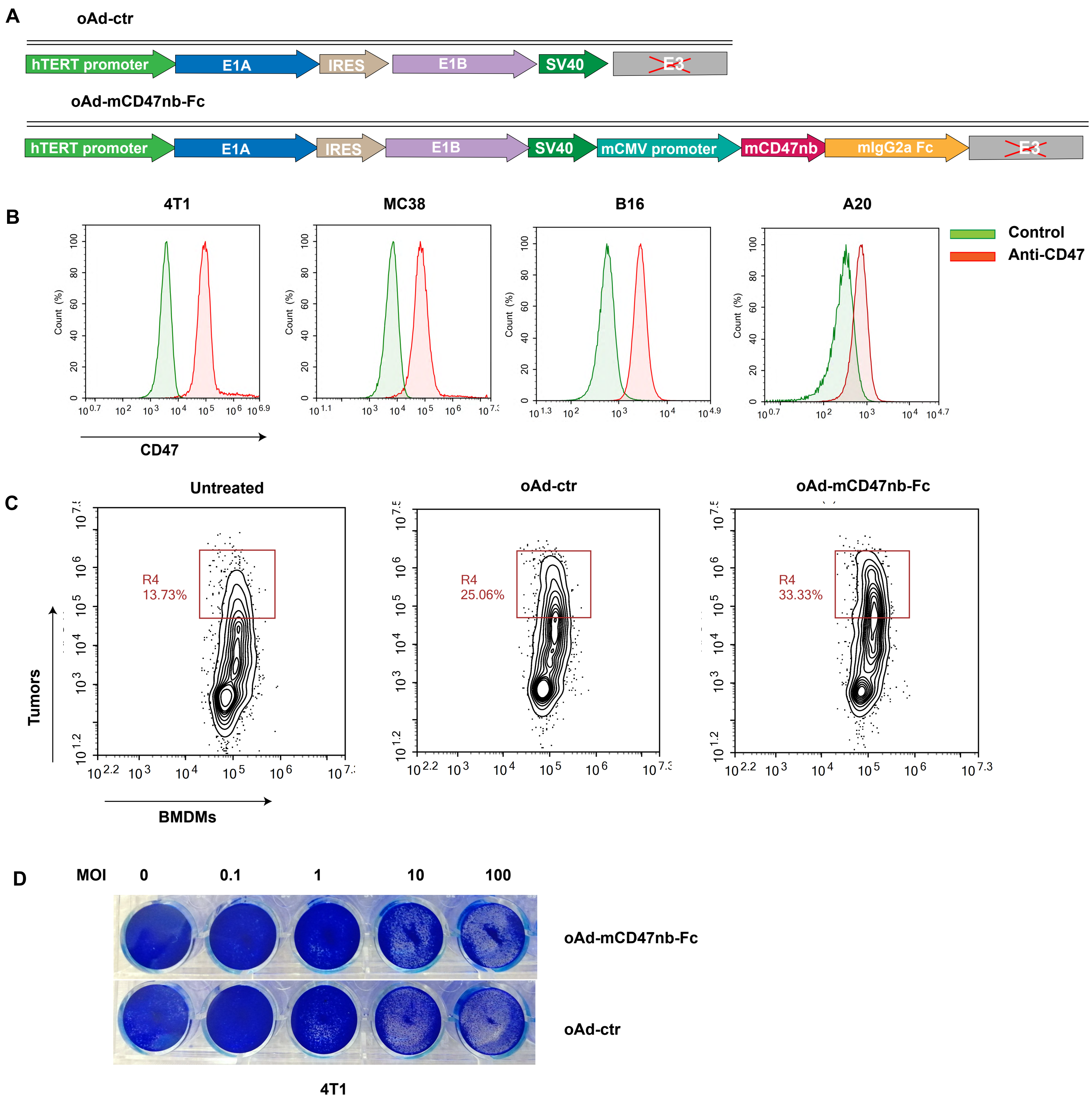

Figure S1. (a) Map of recombinant adenovirus control (oAd-ctr, up) and adenovirus loaded with mCD47nb-Fc transgene (oAd-mCD47nb-Fc, down) used in this study. (b) Flow cytometry images of the expression levels of CD47 on various tumor cell lines examined. (c) Representative flow cytometry images of phagocytosis of 4T1 cells by BMDMs in response to different treatments. Phagocytosis was quantified as the percentage of CFSE-positive macrophages that have engulfed PHK26-positive 4T1 cells.(d) 4T1 cells were infected with oncolytic adenovirus for 4 days at different viral titer followed by Coomassie R-250 staining.

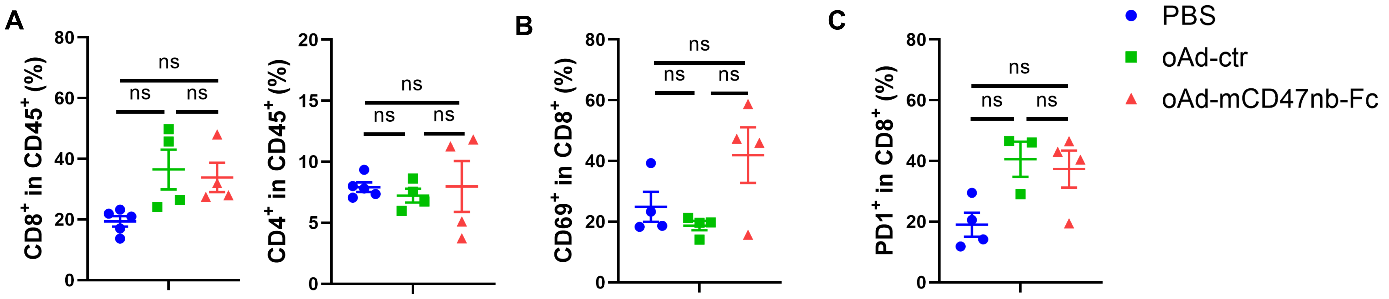

Figure S2. (a) Frequency of CD8+ and CD4+ T lymphocytes within the TME in the B16-F10 melanoma mouse models. (b-c) Analysis of the CD69 expression (b) and PD1 expression (c) of CD8+ T cells of tumors in B16-F10 melanoma-bearing mice.

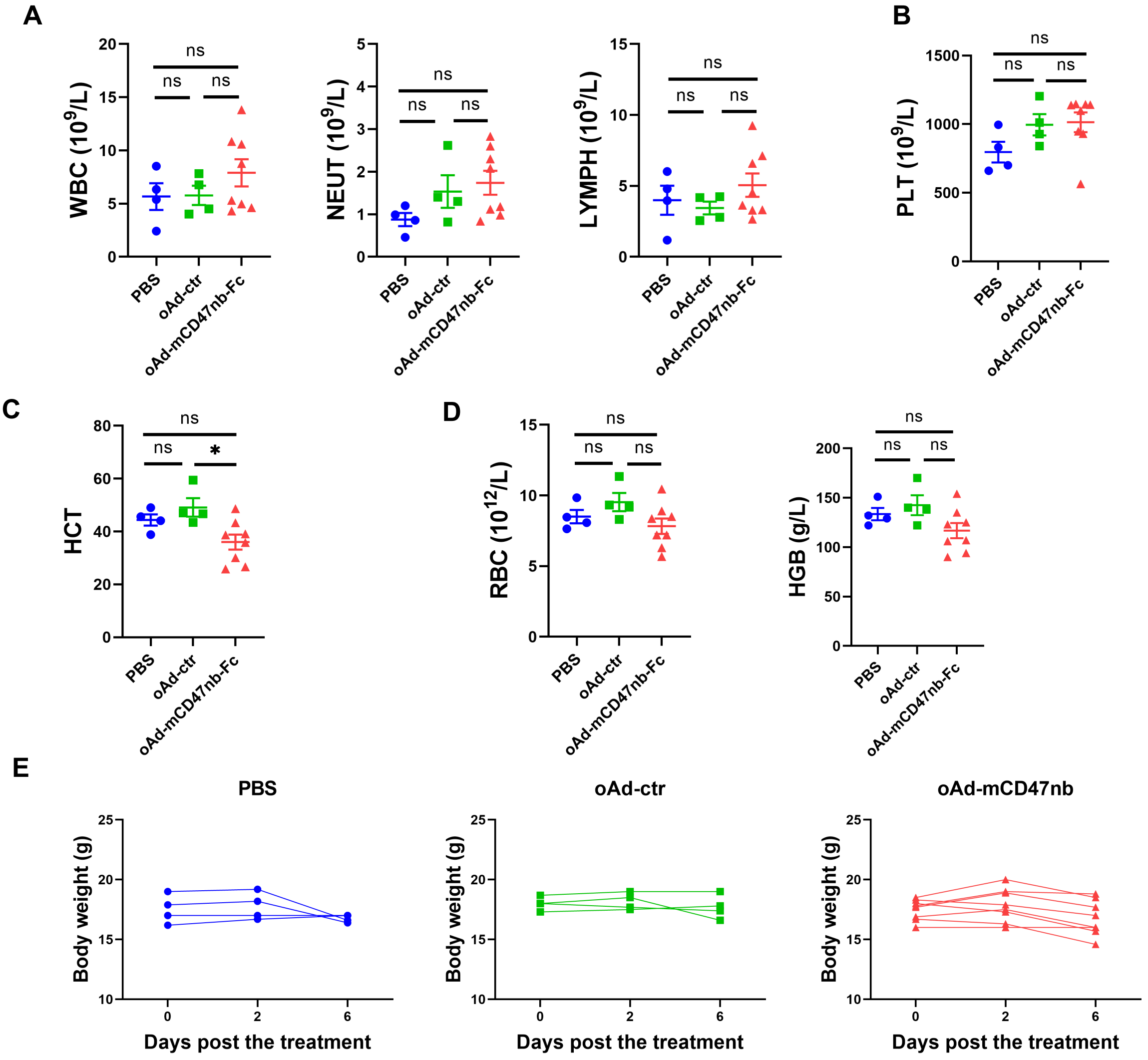

Figure S3 . Local administration of oAd-mCD47nb-Fc circumvents anemia. C57bl/6 mice were implanted with 1×10^6 B16-F10 cells and then intratumorally administered PBS or oncolytic adenoviruses at a dose of 5×10^8 pfu per mouse. Routine blood test was conducted on day 2 after the first administration. (a) Quantification of white blood cells (WBC), neutrophils (NEUT), and lymphocytes (LYMPH). (b) Quantification of platelets (PLT). (c) Hematocrit (HCT) of mice treated with PBS, oAd-ctr, or oAd-mCD47nb-Fc. (d) Quantification of red blood cells (RBC) and hemoglobin (HGB). (e) Changes in the body weight of mice treated with PBS, oAd-ctr, or oAd-mCD47nb-Fc were recorded after treatment. *$P\leq0.05$, ns, not significant ($P>0.05$), all values were compared using one-way ANOVA with Tukey's multiple comparison test. All data are shown as mean ± SEM.
